# Domestic abuse disclosure in perinatal healthcare settings – A qualitative study into the experiences of mothers who had their baby removed at birth

**DOI:** 10.1177/17455057261481007

**Published:** 2026-10-01

**Authors:** Katie Knotts, Abigail Easter, Caroline Bull, Elsa Montgomery, Jane Sandall, Kaat De Backer

**Affiliations:** 1 Department of Women and Children’s Health, Faculty of Life Science and Medicine, 357744 King’s College London, London, UK; 2 Cambridge University Hospitals NHS Foundation Trust, 112009 Rosie Hospital, Cambridge, UK; 3 Methodologies Division, Florence Nightingale Faculty of Nursing, Midwifery and Palliative Care, 14272 King’s College London, London, UK

**Keywords:** child protection, domestic abuse, perinatal healthcare, disclosure, pregnancy

## Abstract

**Background:**

In the UK, numbers of newborn babies entering state care through child protection processes have significantly increased in the last decade. Their mothers often have experienced cumulative and intersecting disadvantages, including childhood abuse and domestic abuse (DA). DA will often be a key factor in child protection decision-making, with a persistent narrative of mothers ‘failing to protect’ their children. Given the profound consequences of DA disclosures and the failure-to-protect discourse that might impact care proceeding outcomes for these mothers, it is important to explore the factors influencing DA disclosure and the complexities that this population faces.

**Objectives:**

To explore the experiences of DA disclosure in perinatal healthcare settings among mothers who had a baby removed at birth; To understand barriers and facilitators that impact DA disclosure in this context.

**Design:**

The MUMS@RISC study was a mixed-methods doctoral research study. This manuscript focuses on the qualitative part of the study, with women who had a baby removed at birth due child protection processes.

**Methods:**

A secondary analysis of narrative interview data from the MUMS@RISC study, using Braun and Clarke’s Reflexive Thematic Analysis. Robust lived experience involvement and trauma-informed approaches were adopted throughout the entire study design.

**Results:**

Interview data from 7 women were included. Three themes were generated, each with sub-themes: 1) Previous adversity leading to current vulnerability; 2) ‘”It’s not that easy” – Navigating, disclosing and leaving an abusive relationship’; and 3) Finding a way out.

**Conclusion:**

Lifelong adversity leading to vulnerability and fear, coupled with system-level barriers, each led to inadequate support and care, impacting DA disclosure among women who have their baby removed at birth and appropriate professional response. Professional understanding about these interconnecting levels of barriers to DA disclosure and the role of coercive control in DA and sensitive routine enquiry about DA is crucial to facilitate opportunities for disclosure.

## Plain Language Summary

In the last ten years in UK, there has been a growing number of newborn babies going into foster care because of child protection concerns. Their mothers often experienced many difficulties, such as childhood abuse and domestic abuse. Domestic abuse will often play a role when child protection agencies and family courts make decisions about whether these mothers can look after their babies. This means that for women, telling someone they experience domestic abuse, can have a serious impact. For that reason, it is important to understand what factors play a role in women’s decision to share or not share their experience of domestic abuse. This study wanted to hear from women who had a baby removed from their care after they gave birth about their experiences, to understand how this played a role in their decision to tell a professional about the domestic abuse they experienced during their pregnancy. An advisory panel of women who had also gone through removal of their baby gave advise on how the study was best designed to make all women feel safe and listened to. We analysed the interviews using Reflexive Thematic Analysis, which means that we looked for themes across the different stories of seven women who took part in the interviews. We identified three main themes, each with sub-themes: 1) Previous adversity leading to current vulnerability; 2) ‘”It’s not that easy” – Navigating, disclosing and leaving an abusive relationship’; and 3) Finding a way out. Women often faced challenges since childhood, which made them vulnerable and fearful to tell professionals about domestic abuse. Even when they told someone, they often did not get the support they needed. Professionals have to better understand the complex lives these women have and ask about domestic abuse in a careful, supporting way.

## Introduction

In many countries, the number of newborn babies entering state care through court-ordered removal (also known as care proceedings) shortly after birth has increased significantly in the previous decade.^1,2^ In the UK, approximately 1 in 208 babies are taken into state care within the first few weeks of their life, also referred to as ‘born into care’,^3^ although rates vary regionally due to factors including poverty and the availability of support resources.^4^ One in four women will experience repeat care proceedings within 10 years of their first baby entering care.^5^ Women often feel lost, vulnerable and suicidal after child removal^6^ and their distress can be further compounded by professional judgement and stigma, depicting them as ‘bad mothers’.^7–9^

In the UK, like in other high-income countries, women from deprived areas and from minoritised communities are disproportionally represented among mothers with child protection agency involvement.^10–12^ In addition, women facing care proceedings are often affected by multiple intersecting disadvantages,^13,14^ including childhood abuse and abuse during their adult relationships.^6^ Recent work by De Backer et al. (2025) also highlighted the complex adversity of mothers known to child protection agencies (CPA). Using maternal mortality surveillance data, the study found that among women who died during or in the year after pregnancy in the UK between 2014 -2022, 65% of women with CPA involvement were subject to domestic abuse compared to 3% of women with no CPA involvement. Women with CPA involvement were also more likely to die from psychiatric causes of death, including suicide and substance use, and homicide, than those without CPA involvement.^9^

In the UK, domestic abuse (DA) is often a key factor in child removal cases, with ‘*Domestic abuse: concerns parent is the victim*’ being the most common factor identified in child removal assessments in the 2024/25 UK Department of Educations ‘Children in Need’ report.^15^ Although victims in their own right, women are often held responsible for DA in care proceedings, with a persistent narrative around ‘failure to protect’.^16^ More women than men have been convicted of ‘failure to protect’, with research showing the narrative of ‘bad mothers’ is further amplified when mothers are deemed unable to protect their children.^17^ To our knowledge, no previous studies have focused on DA disclosure among women who had their newborn baby removed from their care shortly after birth. Given the profound consequences of DA disclosures and the failure-to-protect discourse that might impact care proceeding outcomes for these mothers, it is important to explore the factors influencing DA disclosure and the complexities that this population faces.

In the UK, DA is defined by the Domestic Abuse Act of 2021 and described as ‘abusive behaviour from one person to another when both people are aged over 16 and are personally connected’. ‘Abusive’ behaviour can be physical, emotional, economic, controlling, threatening or sexual.^18^ Coercive control is defined by the UK government as ‘an intentional pattern of behaviour that occurs on two or more occasions, or which takes place over time, for one individual to exert power, control or coercion over another’ and is often a central theme seen in DA.^19^ These coercive behaviours can include isolation, manipulation and humiliation,^20^ and since 2015 coercive and controlling behaviour has been classed as a criminal offence in the UK under the Section 76 Serious Crime Act 2025.^21^ It is estimated that 1.6 million women aged over 16 years old in England and Wales experienced DA in 2023-2024.^22^ For this paper, DA will be used to describe all forms of abuse within relationships and families, including Intimate Partner Violence (IPV) and honour-based abuse, in line with the 2021 Domestic Abuse Act,^18^ and DA disclosure will be defined as disclosing experiences of domestic abuse to health care professionals, both in a hospital-based and community setting.

Research highlights a myriad of socio-economic and relational factors that may impact women’s circumstances to become subject to DA. These include adverse experiences in childhood (ACEs) and low socio-economic status, especially during times of vulnerability in one’s life such as adolescence, young adulthood and the perinatal period.^23–27^ Importantly, detailed focus on these factors in research reflects a system which scrutinises women more intensely and risks ignoring the power of the perpetrator, leaving them ‘unseen’.^28^ Monckton Smith^29^ shifted the focus of DA research from victim to perpetrator behaviour, outlining an eight-stage predictable timeline to homicide which is true regardless of the victims’ prior characteristics. The timeline is characterised by prior abusive behaviour, early relationship escalations and continuing coercive control, which can all exploit victims’ vulnerabilities. Women are aware leaving their relationship would cause a significant escalation in risk. Financial dependency alongside feelings of entrapment can make it difficult for women to leave abusive relationships,^30^ and post-separation abuse is common.^31^

The impact of DA on women’s emotional wellbeing is devastating. Recent figures from the UK have shown that among all domestic-abuse related deaths between 2020-2024, more deaths were caused by DA-related suicides than homicides.^32^ In addition, DA has been associated with further adversities, including low socio-economic status and disability^33^ and can impact the healthcare that women require.^34^ The impact of DA is further amplified when it occurs during the life-changing transition into motherhood, with women exposed to perinatal DA being three times more likely to suffer from mental health conditions than someone with no exposure to abuse,^27^ including postnatal depression.^35,36^ Poor outcomes have also been reported for children who experience DA within family settings, including serious physical and mental illness, low education attainment as well as unemployment and criminal offending in their adult life.^37^

DA disclosure is challenging for many women and it often goes unnoticed and unreported.^38,39^ Mackenzie et al. (2019) describe how women’s ability to state help-seeking claims for DA, as well as recognition of their eligibility for DA support, is limited by the coercive and controlling behaviours of their perpetrator.^40^ This ties in with the wider literature regarding *candidacy* theory, which views people’s eligibility for care and support as an ongoing dynamic process, defined by interactions between individuals and professionals.^41,42^ ‘Identification of candidacy’ is a crucial first step in this process, and considers the individual’s ability to recognise symptoms requiring medical attention and intervention.^41^ However, disclosure is a two-way process, and professionals play a crucial role in facilitating this process of recognition, to create the most opportune and safe circumstances to facilitate DA disclosure.^43^ Mackenzie (2019) provided additional refinements to the concept of candidacy, in particular in the context of DA, whereby women’s self-identification of candidacy can either be *resisted* by themselves, or *refuted* or *depressed* by professionals that are interacting with women.^40^ Previous studies have explored barriers to DA disclosure in healthcare setting in further detail and highlighted fear, perceived stigma and the quality of relationships with professionals all impact disclosure.^44–47^ Healthcare professionals often lack confidence, knowledge and time to sensitively explore DA, particularly to detect more covert signs of DA, such as coercive control^48,49^ and put in place appropriate DA support in line with current guidance.^50^

## Methods

The study was part of a mixed-methods doctoral research study (MUMS@RISC study, NIHR302565) which aimed to investigate the perinatal outcomes and experiences of maternity care among women in contact with child protection services during pregnancy and early motherhood, with quantitative findings already published.^9,51,52^ For the qualitative part of the MUMS@RISC study, a narrative interview study was conducted with eight women who experienced CPA involvement and child removal. The primary narrative analysis has been published elsewhere.^53^ A main study objective in the narrative study was to understand women’s life circumstances prior to pregnancy, to contextualise why CPA had become involved during pregnancy. As a result, the narrative interviews elicited detailed descriptions of experiences of DA among seven of the eight participants in the original study. When exploring these experiences during interviews, the impact of DA on women’s perinatal healthcare, the barriers to access care and DA disclosure became a strong focal point, and, and warranted a further in-depth exploration through thematic analysis. This manuscript contains this secondary analysis of interview data, using Thematic Reflexive Analysis. The reporting of this study conforms to the COREQ statement and is available in the supplementary materials.

### Lived experience involvement and trauma-informed approaches to research participation

At the heart of the MUMS@RISC study was an advisory panel of six women with lived experience of infant removal, representing a range of ethnic, geographical and socio-economic backgrounds. They were consulted at every stage of the research process, with reimbursement for their time and commitment. The ongoing reflections, suggestions and advocacy of the MUMS@RISC advisory panel have shaped the study and this manuscript.

The panel and lead researcher critically reflected on trauma-informed principles throughout the entire research journey, as represented by the MUMS@RISC Charter for Research Engagement (see supplementary materials), with several measures to ensure trauma-informed research participation put in place: 1) Third sector organizations that support women after custody loss provided a safety net pre- and post-interview; 2) A safety protocol was co-designed with the MUMS@RISC advisory panel, to ensure participants’ and researcher wellbeing during or after the interview; 3) Participants were able to have a support person present during the interview; 4) A debrief was provided by the lead researcher (KDB) within 48hrs after the interview, offering an opportunity for informal feedback, further reflection and a safety check-in. 5) Participants were invited to remain involved during the analysis stage, and asked to comment on and sense-check the transcript of their interview, any preliminary analysis, as well as the draft manuscript, and the selection of their individual quotes.

### Recruitment

Women were recruited for the narrative interview study through third-party organisations (MPower, PAC-UK, Foundations, Flourish, Strengthening Families) that support women who have experienced child removal. Women were approached to take part if:

- they were aged over eighteen years old at the time of interview
- they were separated from their infant within six weeks after giving birth due to safeguarding concerns. This includes both court-ordered removals as well as voluntary parental agreements with Children’s Social Care (Children’s Social Care is the term used for Child Protection Agencies in local communities in England.)
- they had received some care by healthcare professionals during the perinatal period
- The separation must have taken place in the last five years
- They could access support through one of the third-party organisations

Women were only approached if their designated key worker in the supporting organisations assessed they would be eligible, to avoid feelings of rejection. In addition, key workers were advised to bear the following exclusion criteria in mind, when approaching women:

- Current (ongoing) involvement in care proceedings in the family court
- Any immediate concerns that the interview process might trigger significant mental health deterioration or trauma responses
- If women were about to be ‘discharged’ from the supporting organisation, and would be unable to access pre-and post-interview support

Informed consent was obtained from all the women, after information was provided to participants regarding the purpose of the study, why they had been approached and the risks of taking part. Written consent was obtained during face-to-face interviews. An online consent form was shared for telephone and online interviews yet at times was difficult to be completed. In these cases, verbal consent was obtained to be inclusive of those with more limited access to technology by reading the consent form aloud, and verbal consent being obtained, audio-recorded, transcribed, and returned back to the participant, in line with the ethical approvals for this study. While recruitment materials did not explicitly state reimbursement fees, women who participated in the study were compensated for their time, either by bank transfer or voucher (£75) and reimbursed for any travel or childcare expenses.

### Data collection

Interview data were collected from October 2023 to November 2024 through narrative interviews, and conducted face-to-face, via video call or via telephone call. A pilot interview was carried out with one of the MUMS@RISC advisory panel members, who met the inclusion criteria. As per her preferences, her interview data was included in the study. Face-to-face interviews were carried out at the participant’s residence or a confidential space in the third sector organisation’s premises. Three women preferred to have their key worker from the support organisation present during the face-to-face interview. Interviews had an average length of 2 hours 15 minutes, and duration ranging from 1 hour 20 minutes to 3 hours 45 minutes. A topic guide (available in supplementary materials) was prepared with a panel of women with lived experience of infant removal, yet was only used to prompt participants if they indicated this would be helpful. Interviews were recorded either through the video call software (where relevant), or a secure recording device, and transcribed by a third-party transcription company. Content warnings regarding the sensitive nature of the audio recordings were shared, to reduce the risk of vicarious trauma among transcribers. Transcripts were checked by two researchers (KK and KDB) and returned to the participants with a thank-you card.

To ensure the safety of the participants, several safety mechanisms were implemented. Firstly, before commencing the interview, contact details of the participant’s key worker were acquired. Secondly immediately after the interview, a debrief took place. Finally, a 48 hour follow up to check on the well-being of the participants. The researcher conducting interviews (KDB) had a clinical academic background as a specialist midwife in perinatal mental health and as such had substantive clinical experience in having difficult yet meaningful conversations with women on a range of difficult topics as well as being trained in trauma-informed interviewing.

### Data analysis

As DA was emerging as a commonality during narrative analysis of the interview data, a secondary analysis was undertaken to explore these experiences in more depth and their impact on healthcare seeking and DA disclosure in perinatal healthcare settings. To this purpose, we conducted a Reflexive Thematic Analysis (RTA) using NVivo 14 software and guided by the six-step framework devised by Braun & Clarke,^54^ see Table 1. We also we adopted Braun and Clarke’s approach to data saturation, i.e. whereby reflexive thematic analysis is not about finding a set number of themes, ‘hiding in the data’, but about interpretation, meaning-making and understanding complexity in the data available.^55^ Rather than *data saturation*, there was *data adequacy*,^55^ *information power*,^56^ *data richness*^55^ and *conceptual depth*.^57–59^ Fieldnotes during the interviews, as well as a log of all versions of the NVivo codebooks and group discussions were kept by the first (KK) and lead researcher (KDB). In addition, the multidisciplinary composition of the research team, with iterative discussions throughout the research cycle regarding theme development, reflexive analysis, interpretation and participant sense-checking all contributed to the rigour of the analysis.

**Table 1.** Braun & Clarke’s six-step framework for reflexive thematic analysis.^54^

Braun & Clarke’s six-step framework for reflexive thematic analysis	
Step 1:	Familiarisation with the data, by thoroughly reading and actively engaging with the transcripts numerous times. Relevant areas of the transcript surrounding the barriers to DA disclosure were identified, annotated and reflections on the data were gathered.
Step 2:	Using NVivo 14 software, transcripts were analysed to generate initial codes. Codes were descriptive and detailed to begin with, and some sections of the transcript were double coded. An iterative approach to coding was adopted, to continuously developing the codebook.
Step 3:	Themes were derived from the data rather than from pre-existing theories. Themes were grouped and re-arranged to create further subthemes. For example: detailed codes such as ‘lost childhood’, ‘lack of freedom’ and ‘loss of identity’ were developed initially. These were grouped together into the parent code ‘internal’ barriers. This was later developed as a subtheme and renamed to ‘Rootless. Voiceless. Powerless’ to encompass all aspects of barriers that women perceived within their own circumstances or life events.
Step 4:	One author (KK) was responsible for coding the data, yet cyclical discussions with the supervisory team allowed for iterative review and adaptation of generated themes, until consensus was reached that the themes were representative of the entire data set.
Step 5:	Re-naming themes appropriately, with the final selection of themes and subthemes.
Step 6:	The write up uses quotations from each of the themes to illustrate findings alongside the pseudonyms selected by the participants. A draft manuscript was shared with all the women who participated in interviews for sense-checking and approval of selected quotations. Five women responded and approved the manuscript in its current state. Two did not respond to the invitation to comment.

### Ethical considerations

This study was approved by the King’s College London Board of Ethics, under the Ethical Clearance Reference Number HR/DP-22/23-35388. All participants consented to participation, chose their own pseudonyms, and any identifying information was removed.

### Positionality and reflexivity statement

The authorship team had a multidisciplinary composition of female clinicians and academics, with a background in midwifery (KDB, CB, JS, EM), psychology (AE)*,* sociology (JS)*,* and medicine (KK). The first author (KK) was a medical student, undertaking an intercalated degree in Women’s Health. CB and KDB both had a clinical background as specialist midwives for women with social complexity, including domestic abuse, substance misuse (CB), mental health problems and learning disability (KDB), with the latter also being an academic researcher who had undertaken a doctoral study in maternity care for women with child protection agency involvement during pregnancy and early motherhood. Through regular discussions, the lead and corresponding author received pastoral support from the wider supervisory team, to mitigate any distressing feelings during the study. Apart from the woman who took part in the pilot interview, participants were not known to the research team prior to recruitment. During the introductory conversations, they were informed that study involvement would not impact (positively or negatively) any contact with child protection agency involvement, or access to support organisations. They were also informed of the lead researcher’s clinical background, her motivation to undertake this research and the overall aims of the MUMS@RISC study. The lead researcher was aware this may have influenced the way in which participants discussed their midwifery experiences.

## Results

This study involved interview data of seven women who had experienced involvement with child protection agencies in the UK and subsequent infant removal and who had been subject to DA. The timing of the removal ranged between 24 hours to six weeks after birth. All seven women were of white ethnicity and were born in England. Women’s ages ranged from 25-41, with an average age of 32.4 years. Four women were single at time of interview, with the other three engaged or in a partnership. At the time of interview, 5 women were unemployed, one was in a full-time employment, and one woman was a carer. Six of the participants were living in rented accommodation, and one had no fixed abode and lived in temporary accommodation. In total the women had given birth to 30 children, with only one child residing in maternal care at the time of the interview. Participant demographics are shown in Table 2.

**Table 2.** Participant demographics.

Demographic characteristics (N=7)	
**Participant’s age (*M***_**Age**_ **=32.4 years)**	
Under 25	-
25-29	n=3
30-34	n=1
35-39	n=2
40 or older	n=1
**Self-reported ethnicity**	
White British	n=7
**Relationship status at time of interview**	
Single	n=4
In partnership	n=2
Engaged/Married	n=1
**Highest educational qualification**	
None	n=3
GCSE or Level 2 BTech (educational qualifications for 16y olds in the UK)	n=2
A Level or Level 3 BTech (educational qualifications for 18y olds in the UK)	n=2
**Employment status**	
Employed	n=1
Carer	n=1
Unemployed, looking for work	n=1
Unemployed, not looking for work (incl. Unable to work)	n=4
**Living arrangements**	
Living alone	n=4
Living with partner	n=3
**Accommodation status**	
Homeless/temporary accommodation	n=1
Privately rented	n=1
Council or housing association rented	n=5
**Parity**	
P3	n=2
P4	n=1
P5	n=4
**Living arrangements of children (N=34)**	
In paternal custody	n=1
In maternal custody	n=1
In kinship care^*^	n=19
In foster placement	n=5
Adopted	n=8

^*^Kinship care represents a legal arrangement where children are placed in the care of relatives, such as a grandparent, aunt or uncle.

Three key themes were generated, each with sub-themes: 1) Previous adversity leading to current vulnerability, 2) ‘It’s not that easy’ – Navigating, disclosing and leaving an abusive relationship, and 3) Finding a way out. An overview of themes and subthemes is presented in Table 3. In the following paragraphs, each of these will be explored, illustrated by relevant quotations, and with pseudonyms chosen by each of the participants.

**Table 3.** Summary of themes and subthemes.

Theme	Subthemes
1) Previous adversity leading to current vulnerability	- Distorted perception of unhealthy relationships - Rootless. Powerless. Voiceless.
2) ‘It’s not that easy’ – Navigating, disclosing and leaving an abusive relationship	- Fear of domestic abuse versus fear of removal - ‘Even if they’re wrong relationships and the bad ones, I still wanted it’ - Professionals turning a blind eye to a black eye - Blaming the victim, whilst empowering the perpetrator
3) Finding a way out	- Lost hope - Finding voice – newfound clarity

### Theme 1 - Previous adversity leading to current vulnerability

This theme captures women’s previous experiences of adversity and abuse, which contributed to a high degree of vulnerability, which was in turn exploited, and thus impacting on women’s views and experiences of DA disclosure. This theme is comprised of two subthemes: ‘Distorted perception of unhealthy relationships’, and ‘Rootless. Powerless. Voiceless.’

#### Distorted perception of unhealthy relationships

This first sub-theme captures women’s desire to find love and stability, through a lifetime of instability and adversity, leading them to seek stability in romantic yet abusive relationships at vulnerable times. However, the formative relationships they had encountered during their childhood and adolescence, either with their parents or boyfriends, tainted their perceptions of what comprises a healthy, loving relationship. This susceptibility to DA was further compounded when they found themselves in situations of extreme vulnerability, like Demi explained.

‘*So then when I was 17 my dad passed away, and my mum kicked me out and I was homeless for a bit […] and I met my first children's dad, who was my domestic abuser.*’ – Demi

Women often described a series of unhealthy relationships from a young age, contributing to this distorted perception:

‘*I’ve been in and out of domestic violence relationships and it’s like a repeated pattern and that goes back to me being about 19 and it just seems to be a consistent pattern of manipulation, financial abuse.*’ – Minnie

For Louise, a similar pattern of recurrent abusive relationships was also present, which she attributed to her difficult childhood and fractured relationship with her own mother.

‘*I think I wanted to be loved. That’s why I couldn’t be without anyone in my life as I grew up. I've always had a boyfriend [….] I jump from relationship to relationship, because I just wanted to be loved by someone and have that, what I didn’t have from my mum.*’ - Louise

Demi attributed her inability to recognise abuse to her childhood experiences of abuse, distorting her understanding of the characteristics of a healthy relationship:

‘*Obviously all I'd ever known was how to be abused, I thought it was a totally normal relationship, I didn't understand it.*’ – Demi

Others had an inkling that their relationship was not healthy, yet were unable to fully comprehend the coercive, controlling and abusive nature of their partner’s behaviour:

‘*I started to see certain little signs but not enough to know that it was actually domestic [abuse].’* - Lizzie

This inability to recognise abusive relationships also meant that women often didn’t realise that support was available or necessary.

#### Rootless. Powerless. Voiceless

Participants described how their experiences of DA and lifelong adversity impacted their ability and readiness to disclose DA. Several women described losing their identity and their voice because of continued demeaning and degrading treatment:

‘*The way I used to be, I was a very, very outgoing […] he turned me into this dark, cold […] just someone who wasn’t me, where I didn’t speak, I didn’t […] It was like, “Yes sir, okay sir, I’ll go and do that, sir”.*’ – Cece

Many women had to grow up quickly after suffering childhood abuse or neglect and described protecting others, such as their siblings:

‘*He would starve us when my mum was away, and my role was to protect, so whatever food he gave us I gave to my siblings, I didn’t want to eat, it was my job to look after them. And then when he tried to beat my brother I got involved, so I got the beating.’* – Daisy

This also meant they never had experienced the freedom to make their own decisions. Women explained how this lack of autonomy continued into adulthood. For several women, it also affected their reproductive freedom, such as Louise, who was forced into a pregnancy and prevented from having an abortion, demonstrating reproductive coercion from her partner:

‘*Then he really wanted a baby, and then he burnt my tablets. He was really controlling’ […] ‘I thought, “It’s not fair bringing two children into the relationship and how it is.” […] I rang the abortion clinic, I booked an abortion appointment, and then somehow, [he] found out. So [he] was ringing me up, threatening me to come to my house, beat the children up and me.’* – Louise

This lack of autonomy and enduring manipulation also meant women needed to protect themselves, and therefore questioned the trustworthiness of others, including professionals:

‘*I lie to people to begin with […] But when I'm starting to work with someone for the long term, I wouldn’t trust them completely first […] I find it hard to trust people.*’ – Louise

### Theme 2 - ‘It’s not that easy’ – Navigating, disclosing and leaving an abusive relationship

This second theme captures the role of professionals and the wider system in the context of DA disclosure. Women described how fear, particularly fear of child removal, was a barrier to initial DA disclosure. For the women in this study, this fear was not irrational, as for many DA and the ‘*failure to protect*’ narrative contributed to the outcome of care proceedings. Professionals often did not understand the complexities of abuse, leading to inadequate professional support. Women described being met with dismissal, a lack of professional curiosity and in some cases, an attitude of victim-blaming. In the following paragraphs, four sub-themes will be further explored.

#### Fear of domestic abuse versus fear of child removal

Fear of the consequences of disclosing DA was highlighted as a barrier by women in this study:

*‘I didn’t let anyone know [I was abused]. Because I was scared.’* – Louise

Participants described being fearful that disclosure would be used against them and would lead to the removal of their children:

‘*We are scared ourselves that you might use it against us. A lot of people don’t want to admit that they’re being abused while being pregnant, because they’re scared their children are going to be removed.*’ – Cece

This highlights a tension between fear of DA and fear of child removal which impacts the women’s decisions to disclose. Demi explained how she only disclosed DA *after* her children were removed. With the children no longer in her care, the fear of them being removed no longer existed, paving the way for disclosure:

‘*I did speak out to the midwife that came to do check-ups, and I was saying ‘look, I'm not coping’. And I think that was because I were more scared that now that I wasn't pregnant, that I was going to get beaten up again.*’ – Demi

#### ‘Even if they’re wrong relationships and the bad ones, I still wanted it’

Women described the complexity of being in an abusive relationship in which they depended on their abusers. One participant described how she knew all too well the relationship was bad but still did not want to leave:

‘*Even if they're wrong relationships and the bad ones, I still wanted it because I never had it as a child.*’ – Louise

Reactions of professionals to DA relationships and disclosure highlight that these complexities are not fully understood:

‘*If they [professionals] can’t end your relationship, they will by taking them babies and if you still don’t end your relationship by taking them babies that should be a clear sign to them that you are in danger, not the children, because who honestly wants to stay in a relationship that’s bad?*’ – Cece

Another complexity captured in this sub-theme pertains to women’s motives to return to their abusive partners after child removal. Feeling isolated and misunderstood, women described finding safety within the familiarity of their partners despite the potential danger:

‘*When you’re going through something with social services and you feel trapped and isolated and that you’re on your own, I was going back to that partner because [pause] that’s where I wanted to be.*’ – Minnie

The perceived support from their abuser was in stark contrast with the lack of professional support and allowed abusers continued power.

#### Turning a blind eye to a black eye

Women described a lack of professional curiosity and urgency during interactions with healthcare professionals. Even when women felt they were exhibiting obvious signs of DA, professionals seemed to turn a blind eye:

‘*I did turn up to contact with a black eye once and no one cared, no one said anything, but in the notes it stated that I turned up unclean, dirty, messy and bruised. That’s a clear sign of a woman that needs help. Massively. But all you did instead of helping me was write it down and use it against me in court*.’ – Cece

They explained how experiences of abuse were dismissed or ignored rather than investigated:

‘*So I went to the doctors, I told them I was being domestically abused… I thought ‘right, I’m going to take this opportunity to try and get some help’, I told the doctor…and he gave me anxiety tablets and sent me out with no help at all.*’ – Demi

These experiences of professional dismissal and limited professional curiosity compounded women’s fear of disclosing DA and created a cycle of fear and hesitance to disclose.

#### Blaming the victim, whilst empowering the perpetrator

This final subtheme captures how professionals seemed to blame women for experiencing abuse whilst empowering the perpetrator. Participants described being punished and villainised for speaking about abuse. Perpetrators were seen as a *‘top shining golden person’* whilst the victim is a *‘villain’, as* Demi described. Professionals appeared to define women by the abuse they experienced, viewing them as beyond help. To make matters worse, women described how previous adversity was being used against them and how they felt they would always be viewed as not having done enough:

‘*And they go ‘right, well we need to take the kid, because you’re being abused’. No, I was, I was abused, I’m a survivor, I’m not a victim no more, I’m a survivor.*’ – Cece

Women described how they struggled to be believed, while their abusers were given more rights and control, even after separation:

‘*Whenever he was in a new relationship, or every time I moved address he'd ring social services up ‘oh, she’s done this, she’s done that, she’s done this’, and none of it would be true, but they'd have to follow it up, and each time they come out they said there was no concerns, everything was fine, they was going to mark it down as malice […] even though they'd mark down it were malice and everything was fine, that then got thrown back at me.*’ – Demi

These experiences put women at further disadvantage. The cycle of being made to feel ashamed and entrapped continued, leaving women feeling abused from every angle:

‘*It’s like you've been abused by your abuser, abused by the system, abused by the social. Nobody wants to help you until it's too late.*’ – Demi

Women shared that even after disclosing DA, they were not viewed as a priority. Limited professional understanding and inappropriate system responses negatively impacted women:

‘*I rang social services the next day, I said, “I'm meant to be going to see my kids today, I've asked to change it to the afternoon,”[…] I said, “I can’t do it. I've just got beaten up. I've been in hospital all night. I cannot […] I need to get some sleep and I need to sort myself out before I see the children.” Then she turned around to me and said, “No.” So social services came out, social services spoke to me […] That’s it. Your contact stops,” because of that reason. So I haven’t seen my kids since then.*’ – Louise

This approach of searching for a quick solution, with a sole focus on the child, rather than getting to the root of the issue and offering both support to mother *and* child was challenged by Cece:

‘*If you know that that woman is in danger whilst she’s pregnant, it’s not just a child that’s at danger. The child is at danger, yes, because she’s in that woman. What you need to do personally is you need to separate it. You need to get involved in this relationship whether you like it or not and rip it apart before it goes too far.*’ – Cece

### Theme 3 - Finding a way out

This final overarching theme captures how women navigated the complex adversities and the system-level barriers they faced. Some women lost hope and felt as though abuse was deserved. In contrast, some women described a turning point which gave them clarity and insights about their situation. Both these opposing perspectives will be explored in the following sub-themes.

#### Lost hope

Ultimately, the barriers experienced by women and inadequate professional responses led women to lose hope and feel like they deserved the abuse they were experiencing.

‘*In the end I just told myself don’t bother asking because you’re not going to get the help you need, so I didn’t bother asking*.’ – Lucy

This lost hope and the emotional burden of abuse led women to display complex emotions towards child removal and DA disclosure. Participants described how they regretted disclosing DA and this contributed to child removal:

*‘If I just shut up and put up, I would have still had my children now.’* – Demi

#### Finding voice – Newfound clarity

Some women described a turning point in their understanding of an abusive relationship and a moment of ‘newfound clarity’:

‘*And when I started to realise, because it's weird, I always say you don't fully mature until you're in your middle 20s, so when I were about 26/27 and I started to realise that things in my childhood wasn't right, and the way I was being treated wasn't right.*’ –Demi

After recognising that their relationship was dominated by abuse, women described feeling empowered and more aware of what a healthy relationship should look like. Many women described being in healthy relationships at the time of interview and being able to take this ‘newfound clarity’ into their future:

‘*I’m not the same girl that I was when I’ve tried fighting back then, I’m now a woman, that’s got a voice, that’s not afraid any more, especially of you [the perpetrator].*’ – Cece

It is important to note that not all women reached this stage of clarity to reflect on past experiences. However, some women described how courses to raise awareness on DA had been beneficial and had increased their understanding. Professional support as well as time away from the perpetrator also enabled clarity:

‘*Going on the domestic courses actually gave me a really, really good insight to make sure that it didn’t happen to me again.*’ – Lizzie

## Discussion

The study aimed to understand the barriers to DA disclosure in perinatal women undergoing care proceedings and identified three overarching themes. Our findings are novel as they explore the perspectives of women who faced infant removal and their experiences of DA disclosure in perinatal healthcare settings. A first theme “*Previous adversity leading to current vulnerability*” explored women’s challenging life courses and self-blame, leading to increased vulnerability for domestic abusive relationships. A second theme, “*It’s not that easy*” identified a myriad of reasons why leaving such abusive relationships is not as straightforward as commonly perceived by professionals or society. A final overarching theme “*Finding a way out*” explored how women were often unable to transition into a more stable, positive place in their life. These findings demonstrate several interconnected barriers to DA disclosure at an individual level, interpersonal level, and at a system-level. Women’s fears and perpetrators’ control play an overarching and cyclical role in connecting the various barriers at each level, whether it is fear of being abused, fear of child removal or fear of disclosing to healthcare professionals. The interplay of these factors contributes to women’s identification of candidacy, i.e the ability to recognise they are in an abusive relationship and their perceived eligibility to request and access support, both by themselves as well as the professionals they encounter.^41,42^

At the individual level, a first significant finding was women’s inability to recognise DA, captured in the subtheme “*Distorted perception of unhealthy relationships*”. Exposure to childhood adversity and unhealthy relationships throughout life shaped women’s perceptions and expectations. Perpetrators exercised coercive control to force women into submission, leaving them unable to fully grasp the harm they were facing and distorting the meaning of love and ‘healthy’ relationships from the outset. These findings echo Stark,^60^ who described how ‘coercive control defers women’s quest for self-realization’ (p.1524). Perpetrator control caused women to internalise blame and their self-blame and guilt made it more difficult to seek help. This thwarted recognition of abusive relationships has previously been reported as a barrier to DA disclosure, particularly when women experience DA in previous relationships.^61,62^ Mackenzie’s (2019) refinements to candidacy theory included this ‘resistance to self-identification’, to describe how women’s ability to self-identify is impaired by the incremental development of abuse, blinding women to the reality of abuse.^40^

A second, individual, barrier to DA disclosure centred on women’s fear, reflected in our subtheme “*Fear of domestic abuse versus fear of removal*”. Similar to Heron and Eisma,^44^ women feared their partner’s reactions, professional judgement and most significantly, the threat of child removal. The latter was not unfounded, as women disclosing DA are known to be at an increased risk of having their children removed.^63^ Conversely, women explained that once child removal occurred, they no longer needed to hide or deny DA, as the fear of child removal that had stopped them from disclosing had turned into reality. This ties in with Mackenzie’s (2019) concept of *unspoken candidacy*, due to fear of child removal,^40^ fear that disclosure might make their situation worse, and/or fear that their candidacy might be refuted.^64^

At an interpersonal level, the role of professionals cannot be underestimated, yet all too often professionals were unaware of the complexities and risk of abuse, and of the coercive control displayed by perpetrators. Inadequate professional responses were captured in our subtheme “*Professionals turning a blind eye to a black eye*”. Our study findings demonstrated a disparity between women’s expectations of DA disclosure and the support they subsequently received. This is in line with previous work describing how women were given mental health advice when disclosing DA, rather than signposting to DA support services, which subsequently led to further internalised shame and suffering.^65^ This inadequate (and inappropriate) response to DA disclosure has been described by Mackenzie (2019) as *depressing* women’s candidacy, whereby professionals’ lack of response ‘matches the silence that abusers try to enforce on their victims’ (p.815).^40^

Despite the statutory requirements around DA enquiry^18^ as well as escalation of ongoing domestic abuse towards child protection agencies,^66,67^ healthcare professionals report that a lack of time and knowledge prevents them from embedding this in their clinical practice.^68–70^ In maternity settings, midwifery staff have reported to feel unprepared to ask about DA, with inadequate knowledge about resources, referral pathways and the wider legal framework around DA^71,72^ Nevertheless, routine DA enquiry is a public health priority.^73^ Most women will find it acceptable to be asked about DA during antenatal enquiry as it makes them aware of any available support.^74^ This is particularly important in the context of women experiencing child removal due to their heightened vulnerability. Unfortunately, most DA assessment tools give more weight to physical incidents, when in fact coercive control is pivotal to identify those subjected to DA yet often under-recognised.^75^ This reflects the lack of professional curiosity which was often experienced by the women in our study.

When women disclose DA to (healthcare) professionals, an appropriate, compassionate response with adequate signposting is essential.^50^ Unfortunately, most women in our sample felt frustrated and disappointed in the way that their DA disclosure was received, particularly regarding the lack of support post-disclosure. As a result, some women described a lack of agency and control over events post-disclosure and felt disclosure had been the wrong decision, reflected by our subtheme “*Lost Hope*”. For some women, the passage of time and access to training courses about DA and coercive control facilitated a clear shift in their mentality and attitude towards their abusive relationships that empowered them to break the cycle of returning to the perpetrator. This was reflected in our subtheme “*Finding voice – newfound clarity*”. Some participants identified domestic abuse awareness courses as helpful, which is in line with evidence suggesting that educational interventions, either in schools or publicly, could be beneficial in improving DA recognition and raising awareness.^76–78^ Unfortunately, such courses are not routinely available, and their preventative value has not been fully explored.

A lack of agency and control was also reflected at a system level, captured by our subtheme of “*Blaming the victim, whilst empowering the perpetrator*”. Our study highlighted that system responses often disadvantaged, blamed and villainised women, in line with previous research findings highlighting that commonly women are held responsible for being with the abuser rather than the responsibility lying with the perpetrator.^16,17,79^ Women’s candidacy claims in the context of abuse are depending on how ‘credible’ they are.^40,41^ Professional attitudes and system responses that discredit women’s credibility (also described as ‘*refuted candidacy’*) will disproportionately affect women who present with vulnerabilities and social risk factors, which further undermine their trustworthiness in health and social care professionals.^40^ Our findings support this and echo the ‘mother-victim-blame’ or ‘failure-to-protect’ narrative seen in health and social care systems and wider society.^80–83^ Such inadequate system responses can impact future help-seeking behaviours and compound women’s fear to disclose.^40^ Furthermore, perpetrators can continue to assert their control by making false claims against their (ex-)partner, thus further contributing to the removal of children and prolonging contact with the perpetrator and continued abuse.^84,85^ This ongoing manipulation and coercive controlling by the perpetrators, can lead to continued isolation, and feeling misunderstood and abandoned by services.^86^

The cyclical reinforcement of women’s fear, through coercive control by their abusive (ex-)partner, and women’s perceived inability to exercise opposition and control transcends all levels of barriers described above. Without adequate recognition of this ongoing pattern, perinatal healthcare settings will be ineffective in detecting signs of coercive control and DA, facilitating disclosure and signposting to appropriate DA support services.

## Strengths and limitations

This study is novel as it seeks the views of women in care proceedings, who are often marginalised in society, healthcare and research. A trauma-informed approach was adopted throughout the entire study design, not only to research participants but also the lived experience panel. An important strength of this paper is that it serves to redress the misrepresentation of women who are subject to DA within previous health research as experiencing isolated incidents of violence from an abstract ‘invisible perpetrator’ for which they are subsequently held accountable for. The study also challenges previous DA health research by highlighting how this narrative can lead to professionals inadvertently colluding with the perpetrator as they fail to understand how the pattern of controlling and coercive tactics serve to obfuscate and hide the daily reality of victim/survivor’s lives to those around them.

Qualitative narrative interviews allowed women to share their stories in detail and great care was taken to provide ongoing support throughout the research cycle, as such recruitment was limited to those with involvement from third-party organisations and those aged 18 or older. However, this study also has some limitations, as experiences may differ from those with no access to support or adolescent girls who are pregnant. Additionally, despite concerted efforts to recruit an ethnically diverse sample, participants were all White British, and our findings might therefore not be transferable to experiences of women from different cultural or ethnic backgrounds, or to those from outside the UK. From discussions with the third sector organisations and community groups for mothers from the Global Majority, it became apparent that women from ethnically and culturally diverse backgrounds may experience an intersectional ‘blind spot’ in third sector support: third sector support for women with custody loss is not consistently available across the UK, and this postcode lottery effect may be further amplified as services may not be culturally adapted to women from diverse backgrounds.

It also has to be acknowledged that some women may have altered their responses in fear of judgment and despite best efforts to minimise bias, this may have impacted the results. Finally, the primary approach for data collection and analysis through narrative methods ensured the focus of this study was participant-led, with experiences of DA emerging as a commonality across the interview data. While the initial interview topic guide did not contain specific questions about DA, further explorative questions were asked where this was appropriate, resulting in rich and detailed interview data. Participant involvement throughout data collection and analysis stages and sense-checking of the manuscript not only has provided an opportunity for participants to retain control of their narrative, but also amplifies the trustworthiness of our findings.

## Implications for clinical practice and future research

This study highlights the need for urgent measures to address the limited awareness surrounding DA that exists amongst women and professionals. This is needed to shift the current focus on violence and move towards and increased understanding of how coercive and controlling behaviours impact women in the perinatal period and form the basis of almost all domestic abusive relationships.^29,87^ In line with the barriers identified in this study, the following recommendations for clinical practice should be at the forefront: Firstly, at the individual, personal level, early educational and awareness interventions should be made easily available to pregnant and postnatal women as well as improved training for professionals, to recognise signs of domestic abuse, including coercive control. Second, at the interpersonal level, our findings highlight the importance of routine DA enquiry and its role in promoting opportunities for disclosure. However, routine DA enquiry needs to recognise the potential repercussions on child protection processes and women’s fear of separation. To facilitate meaningful conversations that foster a safe space for disclosure, professionals must adopt a trauma-informed lens to understand how women’s previous life experiences might impact insight, willingness to disclose, and fears preventing disclosure. Finally, at a system level, trauma-informed approaches in maternity care need to acknowledge the full breadth of trauma, abuse and coercive control that many women with CPA involvement have experienced throughout their life course. Trauma-informed care has become a service improvement priority,^88^ yet may be meaningless if it does not incorporate a detailed understanding of the chronic trauma and enduring consequences that women who are subject to DA are living with daily and how this then impacts their actions, interactions with professionals and behaviour as they navigate the risk to themselves and their children. Their fears must be carefully and sensitively explored, so women feel heard and understood by professionals at an early stage, while ensuring healthcare professionals are not invertedly making it more difficult for women to disclose. When women decide to disclose, improved post-disclosure support is essential to ensure women feel their safety is a priority.

Future research should focus on the role of DA awareness courses in facilitating disclosure and strengthening referral practices to DA support for this population.^89^ In addition, the views and experiences of diverse populations need to be further explored to understand whether and how barriers to disclose differ.

## Conclusion

This study explored the different interconnecting levels of barriers to DA disclosure among women who have their baby removed shortly after birth. Lifelong adversity leading to vulnerability and fear coupled with system-level barriers, each led to inadequate support and care, impacting DA disclosure. Coercive control from perpetrators exploited women’s vulnerabilities and fears, to maintain power and dominance, therefore preventing disclosure. Professional understanding about the role of coercive control in DA and sensitive, routine enquiry about DA is crucial to facilitate opportunities for disclosure. Conversely, negative reactions from professionals and victim-blaming system responses can impact disclosure and exacerbate women’s shame and suffering.

## Supplemental Material

Supplemental Material - Domestic abuse disclosure in perinatal healthcare settings – A qualitative study into the experiences of mothers who had their baby removed after birth Supplemental Material for Domestic abuse disclosure in perinatal healthcare settings – A qualitative study into the experiences of mothers who had their baby removed after birth by Katie Knotts, Abigail Easter, Caroline Bull, Elsa Montgomery, Jane Sandall and Kaat De Backer in Women’s Health

Supplemental Material - Domestic abuse disclosure in perinatal healthcare settings – A qualitative study into the experiences of mothers who had their baby removed after birth Supplemental Material for Domestic abuse disclosure in perinatal healthcare settings – A qualitative study into the experiences of mothers who had their baby removed after birth by Katie Knotts, Abigail Easter, Caroline Bull, Elsa Montgomery, Jane Sandall and Kaat De Backer in Women’s Health

## Data Availability

In view of participant confidentiality and sensitivity of the interview data, data cannot be shared.*
