# Supplementary material for: Domestic abuse disclosure in perinatal healthcare settings – A qualitative study into the experiences of mothers who had their baby removed at birth
Source: Womens Health (Lond). 2026 Oct 1;22:17455057261481007. doi: 10.1177/17455057261481007 (PMC13735634; doi:10.1177/17455057261481007)
Supplement: Supplementary file 1 [file sj-pdf-1-whe-10.1177_17455057261481007.pdf]

Supplementary materials

Supplementary materials 1 The MUMS@RISC Charter for Research engagement

This charter brings together the values that underpin the MUMS@RISC study and we strive to reflect these values in all parts of the research, such as the research design, our interaction with research collaborators and any research communication. The charter was developed by the MUMS@RISC advisory panel, consisting of mothers who experienced removal of their baby after birth and inspired by the Charter for survivor engagement of Survivors' Voices.

- **Respectful, safe and Caring**: A respectful, safe and caring environment is crucial to any research engagement. Everyone is unique with their own story to tell, and we welcome, respect, and celebrate people's diverse backgrounds, experiences, knowledge and skills.

- **Being heard, seen and valued**: We acknowledge that many collaborators will have been on the receiving end of dehumanising processes. Their problems became the only thing that seemed to matter to others and they were no longer seen as a person with a voice and feelings. Our research engagement wants to be person-centered, not problem-centered.

- **Empowering**: We acknowledge that women too often have been silenced and disempowered, with little control of their own narrative. Experts by experience must be empowered to have control over decisions about their own involvement. Research engagement should be positive and give women a voice to express what change they wish to see and explore how they feel this can be achieved.

- **Accountable, transparent and effective**: There is no 'hidden' agenda with this research: all communication should be transparent and clear. Processes and decision-making should be relational, honest, real, transparent and open to feedback and dialogue.

- **Trauma-informed**: We acknowledge that experiences of trauma have shaped the lives of many of research collaborators and participants and that participating in research engagement might trigger some of these traumatic experiences. We strive for a research environment where one feels safe and we aim to avoid such triggers, intentionally and unintentionally. Where required, women can access additional support and safety protocols are in place to ensure the psychological safety of all involved.

- **Poverty-proof:** We acknowledge that the cost-of-living crisis is disproportionately affecting households on lower incomes and that this creates additional obstacles in participating in public life, and by extension research engagement. Any effort will be made to enable research collaboration and avoid 'un-noticed' out of pocket expenses, such as joining meetings online on their phone using their own data, travel costs, etc. Collaborators are able to decide how they would like to be reimbursed for their time based on their own financial circumstances (i.e. transfer, vouchers, combination) and can access advice from a specially trained Citizen Advise Bureau Advisor with expertise in this area.

- **Accessible and inclusive:** Research should be accessible and inclusive to all, and so should the outputs of research. We strive to create accessible, inclusive and appealing research outputs, to amplify the voices of those that participated, while avoiding jargon.

- **Hopeful:** We acknowledge that research collaborators often have lived very difficult lives, marked by trauma, and exacerbated by the grief of the loss of their child(ren). Meaningful research engagement should aim to instil hope for change and a better future and celebrate the contributions of those involved.

- **On this journey together**: None of us are perfect and we jointly learn how to make this process more worthwhile and enduring. There is a joint commitment to continue to listen and learn from one another and to ensure the values in this charter are upheld throughout the entire research journey.

Supplementary materials 2 Topic guides

PART I

In your own time and words and starting however you want, can you please tell me a little bit about your own childhood?

Growing up, as a teenager, what was life like for you?

How did life unfold for you as an adult?

Can you tell me a little bit about what was going on just before you became pregnant?

Looking back on your life, was there anything that you haven't mentioned, and that might have contributed for social services to become involved during your pregnancy?

PART II

How did you find out you were pregnant?

Can you tell me a bit about the initial contact with your midwifery team or your midwife at the beginning of your pregnancy?

Did you have any referrals to services at the beginning of your pregnancy? Can you tell me a bit more about those?

Can you tell me a bit more about what the rest of your pregnancy was like?

When the birth was getting closer, how were you involved in any plans or decisions?

Can you tell me a bit about the care you received during labour and birth?

What were those first moments with your baby like?

Can you tell me a bit about the postnatal care received in the hospital?

Can you explain the circumstances prior to your baby being removed from your care?

If you can, can you tell me a bit about how your baby was removed from your care?

When you were discharged from the maternity hospital, what was the care like from there on?

What was your physical and mental health like in the weeks and months after the birth and removal?

Looking back now, do you understand why your baby was removed?

Did you understand at the time?

Looking back now, what do you think is needed in pregnancy and the postnatal period to help women who go through something similar?

Based on your experience, what does good care look like to you in these circumstances? Bad care?

Finally, is there anything that you want to share, or that I perhaps haven’t asked you but you were expecting me to?

If there is anything else you wanted to add after today, please know you can also get in touch afterwards.
